# Network analysis and relationship of symptom factors to functional outcomes and quality of life following mild traumatic brain injury: a TRACK-TBI study

**DOI:** 10.3389/fneur.2023.1308540

**Published:** 2023-12-12

**Authors:** Shawn R. Eagle, Sonia Jain, Xiaoying Sun, Jonathan Preszler, Michael A. McCrea, Joseph T. Giacino, Geoffrey T. Manley, David O. Okonkwo, Lindsay D. Nelson, The TRACK-TBI Investigators

**Affiliations:** ^1^Department of Neurological Surgery, University of Pittsburgh, Pittsburgh, PA, United States; ^2^Department of Family Medicine and Public Health, University of California, San Diego, San Diego, CA, United States; ^3^Sanford Health, Bismarck, ND, United States; ^4^Medical College of Wisconsin, Milwaukee, WI, United States; ^5^Physical Medicine and Rehabilitation, Harvard University, Cambridge, MA, United States; ^6^Department of Neurosurgery, University of California, San Francisco, San Francisco, CA, United States

**Keywords:** network analysis, mild traumatic brain injuries, trauma, symptoms, quality of life

## Abstract

**Introduction:**

Mild traumatic brain injury (mTBI) is a heterogenous injury which can be difficult to characterize and manage. Using cross-sectional network analysis (NA) to conceptualize mTBI symptoms offers an innovative solution to identify how mTBI symptoms relate to each other. The centrality hypothesis of network theory posits that certain symptoms in a network are more relevant (central) or have above average influence over the rest of the network. However, no studies have used NA to characterize the interrelationships between symptoms in a cohort of patients who presented with mTBI to a U.S. Level 1 trauma center emergency department and how subacute central symptoms relate to long-term outcomes.

**Methods:**

Patients with mTBI (Glasgow Coma Scale = 13–15) evaluated across 18 U.S. Level 1 trauma centers from 2013 to 2019 completed the Rivermead Post-Concussion Symptoms Questionnaire (RPQ) at 2 weeks (W2) post-injury (*n* = 1,593) and at 3 months (M3), 6 months (M6), and 12 months (M12) post-injury. Network maps were developed from RPQ subscale scores at each timepoint. RPQ scores at W2 were associated with M6 and M12 functional and quality of life outcomes.

**Results:**

Network structure did not differ across timepoints, indicating no difference in symptoms/factors influence on the overall symptom network across time. The cognitive factor had the highest expected influence at W2 (1.761), M3 (1.245), and M6 (1.349). Fatigue had the highest expected influence at M12 (1.275). The emotional factor was the only other node with expected influence >1 at any timepoint, indicating disproportionate influence of emotional symptoms on overall symptom burden (M3 = 1.011; M6 = 1.076).

**Discussion:**

Several symptom factors at 2-weeks post-injury were more strongly associated with incomplete recovery and/or poorer injury-related quality of life at 6 and 12 months post-injury than previously validated demographic and clinical covariates. The network analysis suggests that emotional, cognitive, and fatigue symptoms may be useful treatment targets in this population due to high centrality and activating potential of the overall symptom network.

## Introduction

Mild traumatic brain injury (mTBI) continues to be a global public health concern. mTBI is a heterogenous injury across patients with significant variability in both short- and long-term outcomes (1, 2). There is a growing appreciation that mTBI can be linked to long-term consequences. For example, Nelson et al. (3) reported that 53% of mTBI patients evaluated at U.S. Level 1 trauma center emergency departments (EDs) report injury-related functional impairments 1-year post-injury. Functional impairments can have a direct impact on life quality and satisfaction and are often linked to persistent injury-related symptoms, such as psychological distress (3, 4).

Using network analysis (NA) to conceptualize post-mTBI symptoms offers an innovative solution to identify the interrelationships between a unique cohort’s constellation of symptoms (5). In this framework, each node of the network corresponds to a specific symptom, while “edges” refer to the connections between nodes (i.e., symptoms) (6). NA theory suggests that a given pathology can be characterized as a dynamic relationship among active symptoms and produces a network map of the relationships between these symptoms, with the overall goal to identify individual symptoms or symptom clusters with particularly high influence on the overall network (7, 8). The centrality hypothesis of network theory posits that certain symptoms in an individual’s network are more relevant or have above average influence over the rest of the network (9). For example, the “strength” (e.g., total number of connections for a symptom node) and expected influence, can be statistically compared. Researchers have suggested that targeting treatment to these central symptoms may improve overall symptom burden and reduce recovery time by shrinking the size of the network and reducing the role of the patient’s most influential symptoms (5, 9). However, prior to initiating that type of NA (referred to as temporal network analysis) symptom network structure for patients with mTBI needs to be described at serial recovery timepoints.

Due to the reliance on subjective symptom reports to diagnose mTBI (in the absence of a positive head computed tomography [CT] scan), the relationship between mTBI symptoms has been described in a variety of populations and at different timepoints (7, 10–13). These studies typically use a type of factor analysis to understand the factor structure within the instrument, which is useful for identifying latent constructs which cluster together. However, no studies have used network analysis to characterize the interrelationships among symptoms in a cohort of community-acquired patients with mTBI diagnosed at a hospital ED. Moreover, our understanding of how post-acute symptomatology correlates with long-term functional outcomes and quality of life in mTBI patients who sought acute care at an ED is less complete in comparison to other populations (i.e., athletes, patients who first seek care at specialty clinics, etc.) (8, 12, 13). Patients who seek care from U.S. Level 1 trauma center EDs may have unique characteristics (e.g., high prevalence of positive head CT scans) that make findings from other TBI populations nongeneralizable given unique factors contributing to symptoms and long-term outcomes in this subpopulation.

We used network analysis to describe the interrelationship of mTBI symptoms across timepoints in the Transforming Research and Clinical Knowledge in Traumatic Brain Injury (TRACK-TBI) cohort. The primary aim of this study was to describe group-level symptom networks at 2 weeks, 3 months, 6 months, and 12 months post-mTBI, compare changes in network features over time, and identify the most central symptoms at each timepoint. The secondary aim was to evaluate the association of symptom factors at the two-week timepoint with function and quality of life measures at 6- and 12-months post-injury.

## Methods

The current study is a retrospective cohort analysis of prospectively enrolled patients with mTBI who presented to the ED of 18 U.S. Level 1 trauma centers from 2013 to 2018. Participants or their legally authorized representatives provided written informed consent to participate after being approached by a member of the research team in the hospital. The Galveston Orientation and Amnesia Test (GOAT) was used, in part, to determine competency to self-consent. This study was approved for human subjects research by the institutional review board or ethics committee of each enrolling center.

**Figure 1:**
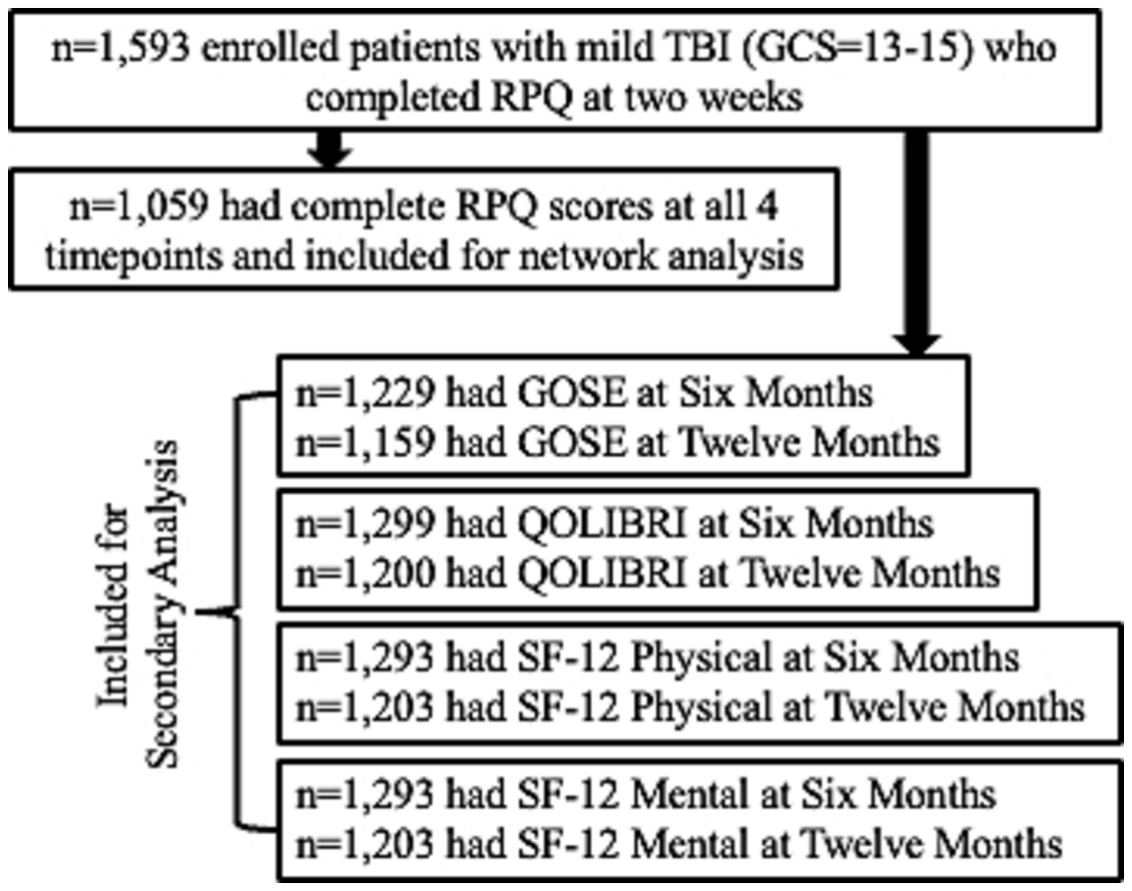
CONSORT diagram.

Participants were included in the study if they presented to the hospital within 24 h of external force trauma to the head meeting the. American Congress of Rehabilitation Medicine’s definition of TBI (14), as well as sufficient for the treating physician to order a head computed tomography (CT) scan. Exclusion criteria included pregnancy, incarceration, nonsurvivable physical trauma, debilitating mental health disorders or neurological disease, magnetic resonance imaging contraindications (for persons in the MRI cohort; e.g., cardiac pacemakers, aneurysm clips, insulin pumps), or any other reason the potential participant could not participate in a longitudinal study. Because the network analysis focused on mTBI, the sample was restricted to patients with a GCS score of 13 to 15 upon ED arrival with complete Rivermead Post-concussion Symptom Questionnaire (RPQ) assessments at 2 weeks (W2), 3 months (M3), 6 months (M6), and 12 months (M12) post-injury (*n* = 1,059). The secondary outcome analysis included GCS 13–15 patients with W2 RPQ and outcome scores M6 and M12 (Figure 1). The Strengthening the Reporting of Observational Studies in Epidemiology [STROBE] reporting guideline.

### Rivermead post concussion symptoms questionnaire (RPQ)

Participants completed the RPQ at W2, M3, M6, and M12. The 16-item RPQ measures severity of headaches, dizziness, and nausea as well as cognitive, mood, and sleep disturbances and other physical symptoms associated with mTBI. Each item is rated on a scale of 0 to 4 within the past 7 days, as compared with pre-injury status, with 0 indicating the symptom was not experienced at all, 1 indicating the symptom is no more of a problem, 2 indicating the symptom is a mild problem, 3 indicating the symptom is a moderate problem and 4 indicating the symptom was a severe problem (15). For analysis, each item was recoded as 0 = no problem or no more of a problem, 1 = mild problem, 2 = moderate problem, and 3 = severe problem the 0 and 1 options in the original RPQ reflect no substantive change in symptom burden.

### Other clinical outcomes

Participants completed a standardized set of outcome assessments at W2, M3, M6, and M12, including the Glasgow Outcome Scale-Extended (GOSE), Short-Form 12 (SF-12), and the Quality of Life after Brain Injury (QOLIBRI-OS) Overall Scale. The QOLIBRI-OS is a health-related quality-of-life instrument used for TBI patients with six items that comprise an overall score (range 0–100, lower scores indicate worse quality of life) (16). The SF-12 is a brief survey of physical and mental health where lower scores correspond to worse self-reported health (17). The Glasgow Outcome Scale-Extended (GOSE) was used to assess functional outcome specific to TBI at W2, M3, M6, and M12 after injury. Complete recovery was defined as a GOSE score = 8; incomplete recovery was defined as GOSE <8.

### Statistical analysis

Descriptive statistics were provided for the analysis cohort. Cross-sectional Gaussian graphical models (GGM) of RPQ symptoms were estimated using the graphical LASSO in combination with EBIC model selection and tuning parameter of 0.5 (18). This procedure required an estimate of a variance–covariance matrix to return a parsimonious network of partial correlation coefficients from which to begin our analyses. Preliminary network analysis revealed strong symptom clusters from the RPQ, where several symptoms (e.g., irritable with depressed and frustrated, poor memory with poor concentration and slow thinking, and blurred vision with light sensitivity and double vision) had uniquely strong relationships (edge weights = 0.29–0.46) with each other. Importantly, these clusters were consistent with prior work by Agtarap et al. (19) which found the symptoms in question formed second-order factors in the emotional, cognitive, and visual domains using exploratory and confirmatory factor analyses. This suggests the symptoms were measuring the same construct, and given network recommendations (20), the individual items within these domains were averaged into three composite variables that reflected Agtarap et al.’s findings (19) and were included as inputs in subsequent network analyses. The emotional composite was an average of irritable, depressed, and frustrated items; the cognitive composite was an average of poor memory, poor concentration, and slow thinking items; and the visual composite was an average of blurred vision, light sensitive, and double vision items. All other individual symptoms were entered as their own inputs.

Network structure was plotted and expected influence of each node was estimated. Expected influence of each node was calculated for each timepoint, which indicates that node’s importance in activating or deactivating other nodes in a network that has negative edges (9). Expected influence scores >1 were considered notable and indicative of high centrality (9).

The Network Comparison Test (NCT: van Borkulo et al. (21)) was used to statistically compare networks from different timepoints. Based on 1,000 permutations, we investigated network structure invariance (possible edge weight differences) and using family-wise Bonferroni corrections. Stability of node strength was evaluated by estimating network models based on subsets of the data using case-dropping bootstrap (6). Correlation Stability (CS) coefficient for correlation values equal or above to *r* = 0.7 were used to measure stability of centrality indices (22, 23). CS-coefficient indicates the percentage of our sample that can be dropped to maintain, with a 95% confidence interval, correlation values (*r*) ≥ 0.7 between our sample’s centrality indices and our bootstrapped samples’ centrality indices (22).

Logistic regression models were used to study the association of W2 RPQ symptom factors with incomplete recovery (GOSE<8 vs. =8) at 6 and 12 months post-injury, adjusting for known risk factors, including demographics (age, sex, years of education), baseline clinical characteristics (prior TBI, psychiatric history), and CT status (positive or negative finding). To reduce the possibility of overfitting and preserve statistical power, symptoms were combined into factors as reported in prior work (19). The decision to analyze factors instead of individual symptoms was also consistent with the network modeling approach described above. In addition to the three factors discussed above, headache, dizziness, and nausea were combined into a somatic factor and noise sensitivity, sleep disturbance, fatigue, and restlessness were combined into an “other” factor. Odds ratio (OR) with 95% confidence interval was reported per unit increase in symptom factors. Similar linear regression models were built for QOLIBRI-OS, and the SF-12 Physical (PCS) and Mental Component scores (MCS). For these continuous outcome measures, beta coefficient with 95% confidence interval was reported. R (version 4.1.2) was used to complete all analyses.^1^

## Results

### Overall cohort

Characteristics of the overall cohort are presented in Table 1. The sample was age 40.6 ± 17.3 years old at time of injury and 66% male. ED arrival GCS was predominately 15 (77%), 18.5% had a GCS = 14, and 4.1% a GCS = 13, with 34.7% having a positive head CT. Only 49.2% of the sample achieved complete recovery (GOSE = 8) at M12.

**Table 1:** Participant characteristics and outcomes.

Age (*n* = 1,593)	40.6 ± 17.3
Male sex (*n* = 1,593)	1,055 (66.2)
Race (*n* = 1,585)	
White	1,224 (77.2)
Black	268 (16.9)
Other	93 (5.9)
Hispanic (*n* = 1,588)	329 (20.7)
Years of education (*n* = 1,560)	13.6 ± 2.9
Psychiatric history (*n* = 1,592)	353 (22.2)
GCS (*n* = 1,593)	
13	65 (4.1)
14	295 (18.5)
15	1,233 (77.4)
CT+ (*n* = 1,550)	538 (34.7)
GOSE at 6 months (*n* = 1,224)	
8	513 (41.9)
7	379 (31.0)
6	230 (18.8)
5	85 (6.9)
≤4	17 (1.4)
GOSE at 12 months (*n* = 1,159)	
8	570 (49.2)
7	319 (27.5)
6	184 (15.9)
5	73 (6.3)
≤4	13 (1.1)
QOLIBRI-OS at 6 months (*n* = 1,299)	66.25 ± 24.76
QOLIBRI-OS at 12 months (*n* = 1,200)	67.58 ± 24.97
SF Physical health at 6 months (*n* = 1,293)	47.40 ± 10.26
SF Physical health at 12 months (*n* = 1,293)	48.08 ± 10.09
SF Mental health at 6 months (*n* = 1,293)	48.46 ± 11.00
SF Mental health at 12 months (*n* = 1,293)	48.79 ± 11.01

Mean ± SD was reported for continuous variables; *N* (%) was reported for categorical variables.

### Comparison of symptom networks over time

**Figure 2:**
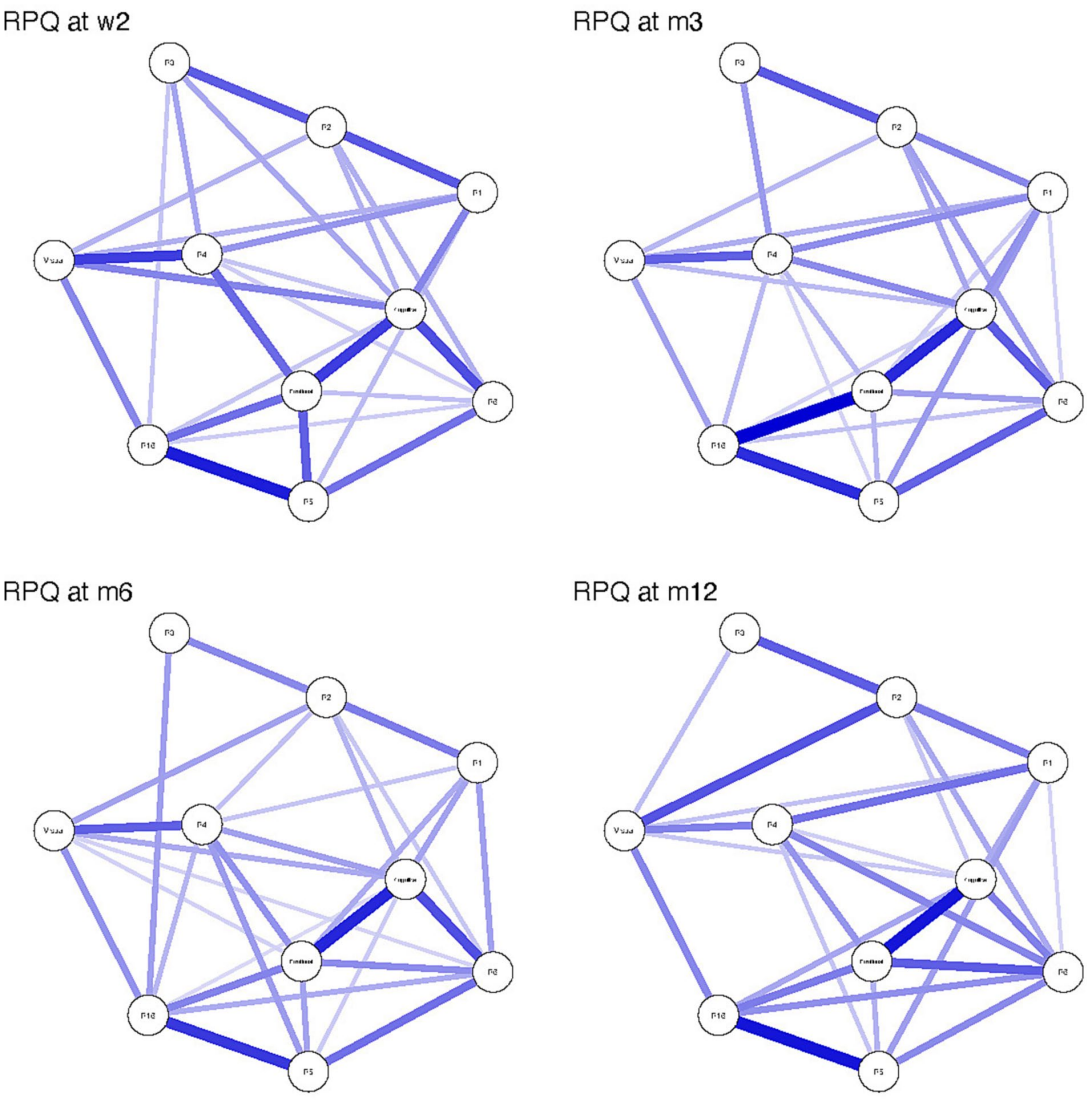
Rivermead Post-concussion Questionnaire (RPQ) symptom network structure at 2 weeks (W2), 3 months (M3), 6 months (M6) and 12 months (M12) following mild traumatic brain injury. Strength of the connection between nodes is illustrated by the thickness and darkness of the connecting edge, where thicker and darker edges mean higher strength of connection. R1 = headache, R2 = dizziness, R3 = nausea, R4 = noise sensitivity, R5 = Sleep disturbance, R6 = fatigue and R16 = restlessness.

Networks over time appear in Figure 2. There was a statistically significant difference in network invariance between the W2 and M12 networks (*p* = 0.026). Post-hoc analysis revealed an absolute edge difference of 0.176 (Bonferroni-Holm corrected *p* = 0.045) between headache and nausea at W2 and M12. There was no statistically significant difference between networks at W2 and M3 (*p* = 0.107); W2 and M6 (*p* = 0.65); W2 and M12 (*p* = 0.13); M3 and M6 (*p* = 0.06); and M6 and M12 (*p* = 0.18). Network strength of edge weights was stable over time, with maximum drop in proportions of 0.517 (W2), 0.595 (M3 and M6), and 0.439 (M12).

Expected influence for each node is presented in Table 2. The cognitive factor had the highest expected influence at W2 (1.761), M3 (1.245) and M6 (1.349). Fatigue had the highest expected influence at M12 (1.275). The emotional factor was the only other node with expected influence >1 at any timepoint (M3 = 1.011; M6 = 1.076). Nausea had the lowest expected influence at all timepoints (−2.007 to −2.363).

**Table 2:** Expected influence values for each node across timepoints.

	Week 2	Month 3	Month 6	Month 12
Emotional	0.885	1.011	1.076	0.790
Cognitive	1.761	1.245	1.349	0.613
Visual	−0.225	−0.868	−0.551	−0.243
Headache	−0.688	0.069	−0.512	0.322
Dizziness	−0.497	−0.434	−0.561	−0.393
Nausea	−2.007	−2.152	−2.065	−2.363
Noise sensitivity	0.276	0.393	0.207	−0.227
Sleep disturbance	−0.157	−0.133	−0.201	−0.331
Fatigue	0.276	0.046	0.597	1.275
Restless	0.375	0.822	0.661	0.558

*a positive expected influence > 1 was suggestive of high centrality.

### Association of symptom factors at 2 weeks with functional and clinical outcomes at 6 months

Regression model outcomes at M6 can be viewed in Tables 3–6. Somatic (aOR = 1.84), other (aOR = 1.55), emotional (aOR = 1.39), and cognitive symptoms (aOR = 1.39) at 2 weeks were significantly associated with incomplete functional recovery (GOSE<8). Emotional (*β* = −5.27), cognitive (*β* = −3.09), other (*β* = −3.58), and visual symptoms (*β* = −3.00) at 2 weeks were significantly associated with lower QOLIBRI-OS scores. Other (*β* = −2.45) and somatic symptoms (*β* = −1.51) at 2 weeks were significantly associated with lower SF-physical scores. Emotional (*β* = −2.72), cognitive (*β* = −1.40), and visual (*β* = −1.51) symptoms at 2 weeks were significantly associated with lower SF-mental scores.

**Table 3:** Logistic regression models predicting incomplete recovery (GOSE<8 vs. =8) at 6 months (left; *n* = 1,172) and 12 months (right; *n* = 1,114) from demographics, head CT findings, and two-week symptoms.

Six months				Twelve months		
	Odds ratio	95% CI	*p*	Odds ratio	95% CI	*p*
Emotional	1.39	1.07–1.81	0.013*	1.08	0.84-1.39	0.551
Cognitive	1.39	1.10–1.77	0.007*	1.72	1.37-2.18	<0.0005*
Visual	1.02	0.73-1.43	0.904	1.33	0.96–1.85	0.088
Somatic	1.84	1.38–2.46	<0.0005*	1.26	0.95-1.66	0.106
Other symptoms	1.55	1.14–2.10	0.005*	1.47	1.09-1.99	0.011*
Age	1.01	1.00-1.02	0.01*	1.01	1.00-1.02	0.003*
Female sex	0.92	0.68–1.24	0.592	1.18	0.87–1.60	0.286
Years of education	0.92	0.88–0.97	0.002*	0.86	0.82-0.91	<0.0005*
Prior TBI	1.13	0.84–1.51	0.426	1.23	0.91–1.65	0.179
Psychiatric history	1.34	0.96–1.86	0.083	1.35	0.97–1.88	0.077
CT+	1.48	1.11–1.97	0.008*	1.73	1.29-2.32	<0.0005*

*statistically significant difference between groups at *p* < 0.05.

**Table 4:** Linear regression models for QOLIBRI-OS at 6 (left; *n* = 1,248) and 12 months (right; *n* = 1,158).

		Six months			Twelve months	
	*β*	95%CI	*p*	*β*	95%CI	*p*
Emotional	−5.27	−7.36, −3.18	<0.0005*	−4.67	−6.90, −2.45	<0.0005*
Cognitive	−3.09	−5.09, −1.10	0.002*	−5.18	−7.24, −3.11	<0.0005*
Visual	−3.00	−5.43, −0.57	0.016*	−0.62	−3.17, 1.93	0.635
Somatic	−1.80	−4.13, 0.53	0.129	−1.74	−4.20, 0.72	0.164
Other symptoms	−3.58	−6.11, −1.04	0.006*	−2.86	−5.53, −0.20	0.035*
Age	−0.20	−0.27, −0.13	<0.0005*	−0.18	−0.25, −0.11	<0.0005*
Female sex	0.64	−1.93, 3.21	0.625	1.69	−1.00, 4.38	0.219
Years of education	0.98	0.56, 1.41	<0.0005*	1.37	0.92, 1.82	<0.0005*
Prior TBI	−3.50	−6.04, −0.96	0.007*	−4.19	−6.86, −1.52	0.002*
Psychiatric history	−10.55	−13.40, −7.70	<0.0005*	−10.06	−13.06, −7.05	<0.0005*
CT+	1.06	−1.48, 3.60	0.413	0.96	−1.69, 3.62	0.476

*statistically significant difference between groups at *p* < 0.05.

**Table 5:** Linear regression model for Short Form 12 Physical Health at 6- (left; *n* = 1,241) and 12-months (right; *n* = 1,161).

		Six months			Twelve months	
	*β*	95%CI	*p*	*β*	95%CI	*p*
Emotional	−0.325	−1.234, 0.583	0.482	−0.300	−1.237, 0.637	0.53
Cognitive	−0.867	−1.732, −0.002	0.05	−1.361	−2.232, −0.491	0.002*
Visual	−0.598	−1.646, 0.451	0.264	−1.064	−2.139, 0.011	0.052
Somatic	−1.510	−2.516, −0.505	0.003*	−0.439	−1.475, 0.596	0.406
Other symptoms	−2.449	−3.542, −1.355	<0.0005*	−2.242	−3.362, −1.122	<0.0005*
Age	−0.185	−0.215, −0.155	<0.0005*	−0.174	−0.204, −0.143	<0.0005*
Female sex	1.094	−0.013, 2.202	0.053	0.624	−0.510, 1.757	0.281
Years of education	0.674	0.491, 0.857	<0.0005*	0.714	0.525, 0.903	<0.0005*
Prior TBI	0.356	−0.739, 1.452	0.524	−0.534	−1.659, 0.592	0.353
Psychiatric history	−2.030	−3.260, −0.799	0.001*	−1.239	−2.503, 0.025	0.055
CT+	2.364	1.271, 3.456	<0.0005*	1.298	0.180, 2.416	0.023*

*statistically significant difference between groups at *p* < 0.05.

**Table 6:** Linear regression model for Short Form 12 mental health at 6- (left; *n* = 1,241) and 12-months (right; *n* = 1,161).

		Six months			Twelve months	
	*β*	95%CI	*p*	*β*	95%CI	*p*
Emotional	−2.715	−3.685, −1.745	<0.0005*	−2.196	−3.238, −1.155	<0.0005*
Cognitive	−1.399	−2.323, −0.475	0.003*	−1.746	−2.714, −0.778	<0.0005*
Visual	−1.510	−2.631, −0.390	0.008*	0.455	−0.740, 1.649	0.456
Somatic	0.109	−0.965, 1.184	0.842	−0.737	−1.888, 0.413	0.209
Other	−0.906	−2.074, 0.263	0.129	−0.597	−1.841, 0.648	0.347
Age	0.009	−0.023, 0.041	0.573	0.012	−0.022, 0.046	0.499
Female sex	−0.836	−2.020, 0.347	0.166	−0.108	−1.368, 1.152	0.866
Years of education	0.081	−0.114, 0.277	0.415	0.357	0.147, 0.566	0.001*
Prior TBI	−1.908	−3.078, −0.737	0.001*	−2.175	−3.426, −0.924	0.001*
Psychiatric history	−5.008	−6.324, −3.693	<0.0005*	−5.004	−6.408, −3.599	<0.0005*
CT+	−0.286	−1.454, 0.881	0.631	−0.260	−1.502, 0.982	0.681

*statistically significant difference between groups at *p* < 0.05.

### Association of symptom factors at 2 weeks with functional and clinical outcomes at 12 months

Regression model outcomes at 12 months can be viewed in Tables 3–6. Cognitive symptoms (aOR = 1.72) and other symptoms (aOR = 1.47) at 2 weeks were significantly associated with GOSE<8. Cognitive (*β* = −5.18), emotional (*β* = −4.67), and other (*β* = −2.86) symptoms at 2 weeks were significantly associated with lower QOLIBRI-OS scores. Other (*β* = −2.24), and cognitive (*β* = −1.36) symptoms at 2 weeks were significantly associated with lower SF-12 Physical scores. Emotional (*β* = −2.20) and cognitive (*β* = −1.75) symptoms at 2 weeks were significantly associated with lower SF-12 Mental scores.

## Discussion

In this study of a large national cohort of participants diagnosed with mTBI at U.S. Level 1 trauma centers, centrality metrics were able to identify that emotional and cognitive symptoms exert an outsized influence on other symptoms at several timepoints. In prior work with this population, Agtarap et al. (19) used exploratory and confirmatory factor analyses to characterize the RPQ and concluded that the RPQ is largely unidimensional. That result was consistent with prior work using a similar model structure with the global symptom checklist from the Sport Concussion Assessment Tool (SCAT) in a population of athletes with acute mTBI (13). Agtarap et al. (19) found that a bifactor model comprising an overarching general factor with three secondary factors (emotional, cognitive and visual) best fit the overall data and remained stable over time. The present study confirms those results, finding no differences in network structure over time and noting strong correlations among the symptoms that comprise the emotional, cognitive, and visual factors. Apart from a slight difference in the relationship between nausea and headache at 2 weeks compared with month 12 of recovery, network structure did not differ across timepoints.

After collapsing the individual symptoms of the above factors for network analyses, the results indicate that cognitive symptoms (i.e., forgetfulness, poor concentration, and taking longer to think) were the most influential to network structure at 2 weeks, 3 months, and 6 months post-injury. Higher expected influence of these nodes suggests that higher symptom scores for this factor could activate connected nodes (Table 2). For example, the cognitive composite was strongly associated with the emotional factor and fatigue (edge weights >0.2; Figure 1) indicating that these symptoms likely co-activate each other. Within the context of a cross-sectional network, which was used in the present study, we cannot determine which symptoms directly cause activation of connected symptoms (i.e., whether emotional symptoms cause cognitive symptoms in this study, or vice versa). Emotional symptoms (i.e., feeling frustrated, irritable, depressed) had slightly lower expected influence scores at 3 and 6 months post-injury but their values likely reflect a similar role within the broader network (Table 2). The centrality of these two symptom factors suggests targeting treatments to these specific domains may “deactivate” the network and reduce overall symptom burden. Future studies need to employ temporal network analyses to delineate cause and effect of early symptom burdens on chronic symptoms, which could provide the basis for identifying useful treatment targets in the ED population with mTBI.

Another key finding from the present study is the principal role subacute symptom burden (i.e., at 2 weeks post-injury) plays in predicting worse functional outcomes and quality of life at 6 and 12 months. Consistently, a single symptom factor carried the first or second strongest association to each long-term outcome, after controlling for known covariates, highlighting the importance of understanding how subacute symptom burden can influence long-term function (24). It is also important to note that certain factors were more relevant to specific long-term domains than others, and this relationship may vary based upon time. For example, somatic symptoms (i.e., headaches, dizziness, nausea) were the primary predictor of incomplete recovery (GOSE<8) and a significant predictor of worse physical health scores at 6 months but were not significantly associated to any other long-term outcome. Pre-injury somatization and high post-injury somatization is a known risk factor for prolonged recovery from sport-related concussion in younger populations (25–27), but less is known about the role of somatic symptoms following mTBI in adults. Nelson et al. found that higher acute somatic symptoms contributed to a prediction model of longer symptom burden for both patients with mTBI and other trauma (28). In a small sample of patients with mTBI recruited from a level 1 trauma center, Stubbs et al. (29) reported higher somatic symptoms in patients with complete recovery (GOSE = 8) than patients with incomplete recovery (GOSE<8). The role of somatic symptoms in recovery from mTBI in adults may warrant future investigation as a potentially modifiable risk factor for recovery at 6 months.

An interesting temporal trend was also observed for the cognitive symptom factor, such that their importance in relation to long-term outcomes seemed to increase from 6 to 12 months. Cognitive symptoms at 2 weeks were significantly associated with higher odds of incomplete recovery, worse quality of life, and worse mental health at 6 months post-injury (Tables 3–6). However, the strength of the association between cognitive symptoms and these outcomes increased at 12 months post-injury. Further, cognitive symptoms went from a non-significant predictor at 6 months to the second strongest predictor of SF-12 Physical health at 12 months (Table 5). Why the association between cognitive symptoms and SF-12 physical health strengthened between six and 12 months post-mTBI is unknown. More research is needed to better understand the role of subjective cognitive complaints following mTBI, especially in relation to long-term outcomes, as cognitive complaints could exist relative to the individual’s prior performance that may not be detectable with neurocognitive testing.

The “other” symptom factor (i.e., sleep disturbance, fatigue, noise sensitivity, and restlessness) was associated with incomplete recovery at 6 (aOR = 1.55) and 12 months (aOR = 1.47) and had the strongest association of all predictors with worse physical health at 6 and 12 months (Table 5). In the network analysis, fatigue at 12 months was the only individual symptom with an expected influence >1 at any timepoint, indicating it may be a useful treatment target. Fatigue is a common symptom following mTBI, occurring in 68% of patients at 1 week post-injury (30), and up to 1 in 3 patients experiences severe fatigue at 6 months post-injury (31). Sleep disturbances are also very common following mTBI, with roughly half of mTBI patients reporting sleep issues and 1 in 4 patients reporting a diagnosed sleep condition (e.g., sleep apnea, insomnia) (32). Despite the prevalence of these symptoms and their relationship to worse outcomes following TBI, very few randomized controlled trials have assessed the utility of interventions targeting these conditions (33). Dischinger et al. (34) reported that endorsing noise sensitivity between 3 and 10 days following mTBI after admission to a level 1 trauma center was associated with 3.1 times higher odds of post-concussion syndrome at 3 months. Noise sensitivity following mTBI appears to be mediated by fear avoidance behaviors (35), which are a possible treatment target for this population but little to no interventional studies addressing this issue exist (36).

There are limitations to this study. Symptoms by nature are self-reported and subject to bias. The networks presented are cross-sectional, so temporal relationships between symptoms could not be investigated. As such, no “cause and effect” conclusions could be made. The symptom factors were correlated with moderate strength (*r* = 0.48 to 0.70). This is common in mTBI symptom assessments due to the commonality of a pervading “general” symptom factor across these tools (12, 13, 19). As a result, the factors are not completely discrete from each other and significant relationships found between individual factors and other outcomes may not be exclusively related to the individual factor. These results are only generalizable to adult patients with mTBI who presented to a level 1 trauma center emergency department. It is not possible to discern the role of any potential treatments on the group-level networks presented, but future research quantifying the temporal effects of targeted treatments on network structure will be important.

## Conclusion

In this longitudinal cohort study of patients diagnosed with mTBI at a level 1 trauma center emergency department, network analysis revealed emotional and cognitive symptom factors had the highest influence on the overall network from the subacute to chronic recovery periods. The network analysis suggests that emotional and cognitive symptoms may be useful treatment targets in this population due to high centrality and activating potential of the overall network (i.e., high expected influence). This finding needs to be validated by a temporal network analysis to determine cause and effect of these symptoms on later individual symptoms and overall network strength. When analyzed as symptom factors, reporting higher symptoms at 2 weeks were associated with higher odds of incomplete recovery and poorer quality of life at 6 and 12 months.

## Data availability statement

The original contributions presented in the study are included in the article/Supplementary material, further inquiries can be directed to the corresponding author.

## Ethics statement

The studies involving humans were approved by the ethics committee of each institution (the full list is available as a Supplementary file). The studies were conducted in accordance with the local legislation and institutional requirements. The participants provided their written informed consent to participate in this study.

## Author contributions

SE: Conceptualization, Methodology, Writing – original draft, Writing – review & editing. SJ: Data curation, Formal analysis, Methodology, Writing – review & editing. XS: Data curation, Formal analysis, Methodology, Writing – review & editing. JP: Methodology, Writing – review & editing. MM: Investigation, Methodology, Project administration, Resources, Supervision, Writing – review & editing. JG: Investigation, Methodology, Project administration, Resources, Supervision, Writing – review & editing. GM: Conceptualization, Data curation, Funding acquisition, Investigation, Methodology, Project administration, Resources, Supervision, Writing – review & editing. DO: Conceptualization, Data curation, Funding acquisition, Investigation, Methodology, Project administration, Resources, Supervision, Writing – review & editing. LN: Conceptualization, Methodology, Project administration, Writing – review & editing.

## The TRACK-TBI investigators

Neeraj Badjatia, MD, University of Maryland; Ann-Christine Duhaime, MD, MassGeneral Hospital for Children; Shankar Gopinath, MD, Baylor College of Medicine; Ramesh Grandhi, MD, University of Utah; Christopher Madden, MD, UT Southwestern; Randall Merchant, PhD, Virginia Commonwealth University; Pratik Mukherjee, MD PhD, University of California, San Francisco; Claudia Robertson, MD, Baylor College of Medicine; David Schnyer, PhD, UT Austin; Sabrina R. Taylor, PhD, University of California, San Francisco; John K. Yue, MD, University of California, San Francisco; Ross Zafonte, DO, Harvard Medical School.

## Footnotes

^1^ http://www.r-project.org

## References

[1] Brett BL Kramer MD Whyte J McCrea MA Stein MB Giacino JT. Latent profile analysis of neuropsychiatric symptoms and cognitive function of adults 2 weeks after traumatic brain injury: findings from the TRACK-TBI study. JAMA Netw Open. (2021) 4:e213467. doi: 10.1001/jamanetworkopen.2021.3467, PMID: 33783518 PMC8010589

[2] Levin HS Temkin NR Barber J Nelson LD Robertson C Brennan J. Association of sex and age with Mild Traumatic Brain Injury-Related Symptoms: a TRACK-TBI study. JAMA Netw Open. (2021) 4:e213046. doi: 10.1001/jamanetworkopen.2021.3046 33822070 PMC8025125

[3] Nelson LD Temkin NR Barber J Brett BL Okonkwo DO MA MC. Functional recovery, symptoms, and quality of life 1 to 5 years after traumatic brain injury. JAMA Netw Open. (2023) 6:e233660. doi: 10.1001/jamanetworkopen.2023.3660 36939699 PMC10028488

[4] McCrea MA Giacino JT Barber J Temkin NR Nelson LD Levin HS. Functional outcomes over the first year after moderate to severe traumatic brain injury in the prospective, longitudinal TRACK-TBI study. JAMA Neurol. (2021) 78:982–92. doi: 10.1001/jamaneurol.2021.2043, PMID: 34228047 PMC8261688

[5] Iverson GL. Network analysis and precision rehabilitation for the post-concussion syndrome. Front Neurol. (2019) 10:489. doi: 10.3389/fneur.2019.00489, PMID: 31191426 PMC6548833

[6] Epskamp S Maris G Waldorp LJ Borsboom D. Network psychometrics In: Irwing P Booth T Hughes DJ, editors. The Wiley handbook of psychometric testing: a multidisciplinary reference on survey, scale and test development. New Jersey: Wiley Blackwell (2018). 953–86.

[7] Iverson GL Jones PJ Karr JE Maxwell B Zafonte R Berkner PD. Network structure of physical, cognitive, and emotional symptoms at preseason baseline in student athletes with attention-deficit/ hyperactivity disorder. Arch Clin Neuropsychol. (2020) 3:acaa030. doi: 10.1093/arclin/acaa030 32619228

[8] Iverson GL Jones PJ Karr JE Maxwell B Zafonte R Berkner PD. Architecture of physical, cognitive, and emotional symptoms at preseason baseline in adolescent student athletes with a history of mental health problems. Front Neurol. (2020) 11:175. doi: 10.3389/fneur.2020.00175, PMID: 32265822 PMC7100766

[9] Robinaugh DJ Millner AJ McNally RJ. Identifying highly influential nodes in the complicated grief network. J Abnorm Psychol. (2016) 125:747–57. doi: 10.1037/abn0000181, PMID: 27505622 PMC5060093

[10] Eagle SR Kontos AP Collins MW Mucha A Holland CL Edelman K. Targeted intervention improves symptoms and impairments in patients with mild traumatic brain injury with chronic symptom: a prospective, multiple interventional research trial. J Spec Oper Med. (2021) 21:61–6. doi: 10.55460/AEY2-8NRI, PMID: 34105123

[11] Eagle SR Womble MN Elbin R Pan R Collins MW Kontos AP. Concussion symptom cutoffs for identification and prognosis of sports-related concussion: role of time since injury. Am J Sports Med. (2020) 48:2544–51. doi: 10.1177/0363546520937291, PMID: 32693612

[12] Kontos AP Elbin R Schatz P Covassin T Henry L Pardini J. A revised factor structure for the post-concussion symptom scale: baseline and postconcussion factors. Am J Sports Med. (2012) 40:2375–84. doi: 10.1177/0363546512455400, PMID: 22904209

[13] Nelson LD Kramer MD Patrick CJ McCrea MA. Modeling the structure of acute sport-related concussion symptoms: a bifactor approach. J Int Neuropsychol Soc. (2018) 24:793–804. doi: 10.1017/s1355617718000462, PMID: 30079858 PMC6589835

[14] Kay T Harrington D Adams R. Mild traumatic brain injury committee of the head injury interdisciplinary special interest group of the American congress of rehabilitation medicine. Definition of mild traumatic brain injury. J Head Trauma Rehabil. (1993) 8:74–85. doi: 10.1097/00001199-199309000-00009

[15] Eyres S Carey A Gilworth G Neumann V Tennant A. Construct validity and reliability of the Rivermead post-concussion symptoms questionnaire. Clin Rehabil. (2005) 19:878–87. doi: 10.1191/0269215505cr905oa, PMID: 16323387

[16] von Steinbuechel N Wilson L Gibbons H Muehlan H Schmidt H Schmidt S. QOLIBRI overall scale: a brief index of health-related quality of life after traumatic brain injury. J Neurol Neurosurg Psychiatry. (2012) 83:1041–7. doi: 10.1136/jnnp-2012-302361, PMID: 22851609

[17] Ware J Jr Kosinski M Keller SD. A 12-item short-form health survey: construction of scales and preliminary tests of reliability and validity. Med Care. (1996) 34:220–33. doi: 10.1097/00005650-199603000-00003 8628042

[18] Foygel R Drton M. Extended Bayesian information criteria for Gaussian graphical models. Adv Neural Inf Proces Syst. (2010) 23:2020–8.

[19] Agtarap S Kramer MD Campbell-Sills L Yuh E Mukherjee P Manley GT. Invariance of the Bifactor structure of mild traumatic brain injury (mTBI) symptoms on the Rivermead Postconcussion symptoms questionnaire across time, demographic characteristics, and clinical groups: a TRACK-TBI study. Assessment. (2021) 28:1656–70. doi: 10.1177/1073191120913941, PMID: 32326739 PMC7584771

[20] Fried EI Cramer AO. Moving forward: challenges and directions for psychopathological network theory and methodology. Perspect Psychol Sci. (2017) 12:999–1020. doi: 10.1177/1745691617705892, PMID: 28873325

[21] Van Borkulo C Epskamp S Milner A. Network comparison test: permutation-based test of differences in strength of networks. (2015). Available at: https://cran.rproject.org/web/packages/NetworkComparisonTest/NetworkComparisonTest.pdf

[22] Epskamp S Fried E. (2019). Bootnet: bootstrap methods for various network estimation routines (R package version 1.2). Available at: https://cranr-projectorg/web/packages/bootnet/bootnet.pdf

[23] Epskamp S Rhemtulla M Borsboom D. Generalized network psychometrics: combining network and latent variable models. Psychometrika. (2017) 82:904–27. doi: 10.1007/s11336-017-9557-x 28290111

[24] Zahniser E Nelson LD Dikmen SS Machamer JE Stein MB Yuh E. The temporal relationship of mental health problems and functional limitations following mTBI: a TRACK-TBI and TED study. J Neurotrauma. (2019) 36:1786–93. doi: 10.1089/neu.2018.6172, PMID: 30543138 PMC6551992

[25] Green KE Purtzki J Chapman A Oberlander TF Silverberg ND Dhariwal AK. Somatization in adolescents with persistent symptoms after concussion: a retrospective chart review. J Neuropsychiatry Clin Neurosci. (2022) 34:378–85. doi: 10.1176/appi.neuropsych.21070169, PMID: 35414192

[26] Nelson LD Tarima S LaRoche AA Hammeke TA Barr WB Guskiewicz K. Preinjury somatization symptoms contribute to clinical recovery after sport-related concussion. Neurology. (2016) 86:1856–63. doi: 10.1212/WNL.0000000000002679, PMID: 27164666 PMC4873681

[27] Root JM Zuckerbraun NS Wang L Winger DG Brent D Kontos A. History of somatization is associated with prolonged recovery from concussion. J Pediatr. (2016) 174:39–44.e1. doi: 10.1016/j.jpeds.2016.03.020 27059916 PMC4925238

[28] Nelson LD Furger RE Ranson J Tarima S Hammeke TA Randolph C. Acute clinical predictors of symptom recovery in emergency department patients with uncomplicated mild traumatic brain injury or non-traumatic brain injuries. J Neurotrauma. (2018) 35:249–59. doi: 10.1089/neu.2017.4988, PMID: 29017409 PMC5784791

[29] Stubbs JL Green KE Silverberg ND Howard A Dhariwal AK Brubacher JR. Atypical somatic symptoms in adults with prolonged recovery from mild traumatic brain injury. Front Neurol. (2020) 11:43. doi: 10.3389/fneur.2020.00043, PMID: 32117012 PMC7010927

[30] Norrie J Heitger M Leathem J Anderson T Jones R Flett R. Mild traumatic brain injury and fatigue: a prospective longitudinal study. Brain Inj. (2010) 24:1528–38. doi: 10.3109/02699052.2010.531687 21058899

[31] Stulemeijer M van der Werf S Bleijenberg G Biert J Brauer J Vos PE. Recovery from mild traumatic brain injury: a focus on fatigue. J Neurol. (2006) 253:1041–7. doi: 10.1007/s00415-006-0156-5 16708266

[32] Mathias J Alvaro P. Prevalence of sleep disturbances, disorders, and problems following traumatic brain injury: a meta-analysis. Sleep Med. (2012) 13:898–905. doi: 10.1016/j.sleep.2012.04.006, PMID: 22705246

[33] Sullivan KA Blaine H Kaye S-A Theadom A Haden C Smith SS. A systematic review of psychological interventions for sleep and fatigue after mild traumatic brain injury. J Neurotrauma. (2018) 35:195–209. doi: 10.1089/neu.2016.4958, PMID: 28895488

[34] Dischinger PC Ryb GE Kufera JA Auman KM. Early predictors of postconcussive syndrome in a population of trauma patients with mild traumatic brain injury. J Trauma Acute Care Surg. (2009) 66:289–97. doi: 10.1097/TA.0b013e3181961da2 19204499

[35] Faulkner JW Snell DL Shepherd D Theadom A. Turning away from sound: the role of fear avoidance in noise sensitivity following mild traumatic brain injury. J Psychosom Res. (2021) 151:110664. doi: 10.1016/j.jpsychores.2021.110664, PMID: 34749069

[36] Callahan ML Lim MM. Sensory sensitivity in TBI: implications for chronic disability. Curr Neurol Neurosci Rep. (2018) 18:1–8. doi: 10.1007/s11910-018-0867-x 30008147

