# Supplementary material for: Network analysis and relationship of symptom factors to functional outcomes and quality of life following mild traumatic brain injury: a TRACK-TBI study
Source: Front Neurol. 2023 Dec 12;14:1308540. doi: 10.3389/fneur.2023.1308540 (PMC10750770; doi:10.3389/fneur.2023.1308540)
Supplement: Supplementary file 1 [file Table_1.docx]

Study Sites:

1. University of California, San Francisco
2. University of Pittsburgh
3. University of Pennsylvania
4. Baylor College of Medicine
5. University of California, San Diego
6. University of Washington
7. Harvard Medical School
8. University of Cincinnati
9. University of Miami
10. University of Texas- Austin
11. Medical College of Wisconsin
12. University of Utah
13. University of Texas Southwestern
14. Virginia Commonwealth University
15. Emory University
16. Indiana University
17. University of Southern California
18. University of Texas- Houston
